# Association between composite dietary antioxidant index and migraine in American young women: insights from NHANES 1999–2004 cross-sectional data

**DOI:** 10.3389/fneur.2024.1399916

**Published:** 2024-09-09

**Authors:** Zeyan Li, Xinyu Zhang, Simin Kong, Chuan-Chuan Fu, Tian-Qi Lv, Bin Xiao

**Affiliations:** ^1^Department of Plastic Surgery, BOE Hospital, Chengdu, China; ^2^Department of Neurology, The First Affiliated Hospital of Harbin Medical University, Harbin, China

**Keywords:** migraine, composite dietary antioxidant index, NHANES, cross-sectional study, dietary intake

## Abstract

**Background:**

Excessive oxidative stress is one of the key pathophysiological mechanisms underlying migraine, and increasing antioxidant intake has proven to be an effective strategy for the prevention and improvement of migraine symptoms. To explore the relationship between the composite dietary antioxidant index (CDAI) and the occurrence of migraine attacks.

**Methods:**

Cross-sectional data from the National Health and Nutrition Examination Survey (NHANES) spanning 1999–2004 were utilized. Logistic regression, stratified analysis, and restricted cubic spline (RCS) models were employed to investigate the association between CDAI and migraine attacks.

**Results:**

A total of 8,137 adults aged ≥20 were enrolled, comprising 1,610 patients with migraine and 6,527 non-migraine individuals. After adjusting for all covariates, CDAI was negatively correlated with migraine. In the overall participants, compared with the CDAI Q1 (−5.83 to −2.14) group, the adjusted odds ratio (OR) for migraine in Q3 (−0.59 to 1.53) and Q4 (1.53–44.63) groups were 0.71 [95% confidence interval (95% CI): 0.54–0.92, *p* = 0.011] and 0.64 (95% CI: 0.47–0.87, *p* = 0.005), respectively. After stratifying by age and gender, the protective effect was more pronounced in females aged 20–50, with adjusted OR for Q3 (−0.59 to 1.53) and Q4 (1.53–44.63) groups of 0.60 (95% CI: 0.40–0.90, *p* = 0.013) and 0.48 (95% CI: 0.30–0.78, *p* = 0.003), respectively. The RCS curve indicated a nonlinear relationship between CDAI and migraine in females aged 20–50, with a threshold of 0.006.

**Conclusion:**

CDAI is negatively correlated with migraine attacks, and a higher CDAI may be an effective protective factor in preventing migraine attacks, especially in women aged 20–50.

## 1 Introduction

Migraine is characterized by episodic headaches with features like sensitivity to light, sound, or motion. It is also recognized as a recurrent headache syndrome with additional neurological symptoms (1). Between 1990 and 2019, the global age-standardized prevalence of migraines increased by 1.7% (2). Migraines are globally the second most common cause of disability. They are most prevalent among young women (3). Beyond severe headaches and disability, patients frequently face considerable psychological and economic burdens. Reports indicate that in the United States, migraine patients bear an economic burden more than double that of individuals without migraines. Post COVID-19, migraine has evolved from a singular condition to a complex issue with multifaceted factors (4). Nonetheless, the treatment of migraines remains challenging due to complex pathophysiological mechanisms. Notably, many studies now emphasize the role of excessive oxidative stress (OS) in migraines (5).

The imbalance between antioxidant and pro-oxidant indicators is considered OS. When the body is under intense OS, the mitochondria generate a substantial amount of reactive oxygen species (ROS) and other byproducts. The excessive accumulation of these substances disrupts and damages various cellular components and normal biochemical pathways uncontrollably. This makes the cells more susceptible to the impact of oxidative compounds in the environment, leading to a reduction in antioxidant defense capability. For instance, ROS can induce migraine through both direct and indirect pathways. The excessive accumulation of ROS can trigger cell apoptosis by inducing cellular autophagy, disrupting normal mitochondrial function and membrane potential balance. Furthermore, it promotes the release of migraine mediators, such as calcitonin gene-related peptide, through various pathways, leading to the disruption of brain tissue and the onset of migraine (6–9). Ultimately, this imbalance damages crucial tissues such as muscles, bones, the brain, and the nervous system (5, 10, 11). It is noteworthy that migraine is closely associated with the level of OS. OS may indirectly impact migraines by affecting their triggers. According to Fila et al., migraine-related OS may increase DNA damage in neurons, astrocytes, and glial cells. Impaired DNA repair in these cells could result in various pathological changes in the brain (6). This influence is also reflected in diseases, such as worsening obstructive sleep apnea leads to a notable decrease in serum albumin, a blood marker with substantial antioxidant activity. Decreased antioxidant capacity in obstructive sleep apnea, exacerbating oxidative stress, can lead to cardiovascular and metabolic abnormalities (12). Therefore, maintaining a balance in normal oxidative metabolism is crucial. Recent views suggest that increasing dietary antioxidant intake holds promise for migraine sufferers (13, 14). This dietary approach, potentially more accessible and acceptable than conventional treatments, aligns with current sustainable development goals, including reducing migraine-related economic and medication burdens (15).

Although previous several studies have assessed the impact of dietary antioxidant intake on migraine (16, 17), reports on the relationship between the composite dietary antioxidant index (CDAI) and migraine are scarce. Therefore, a cross-sectional study was designed based on 1999–2004 data from the National Health and Nutrition Examination Survey (NHANES) database. CDAI, a valuable score confirmed to be associated with various diseases or health issues was introduced (14, 18, 19). A CDAI incorporating six dietary antioxidants (vitamins A, C, and E, carotenoid, zinc, and selenium) was designed for data analysis, to provide a novel and feasible reference for migraine prevention and treatment.

## 2 Methods

### 2.1 Study population

**Figure 1:**
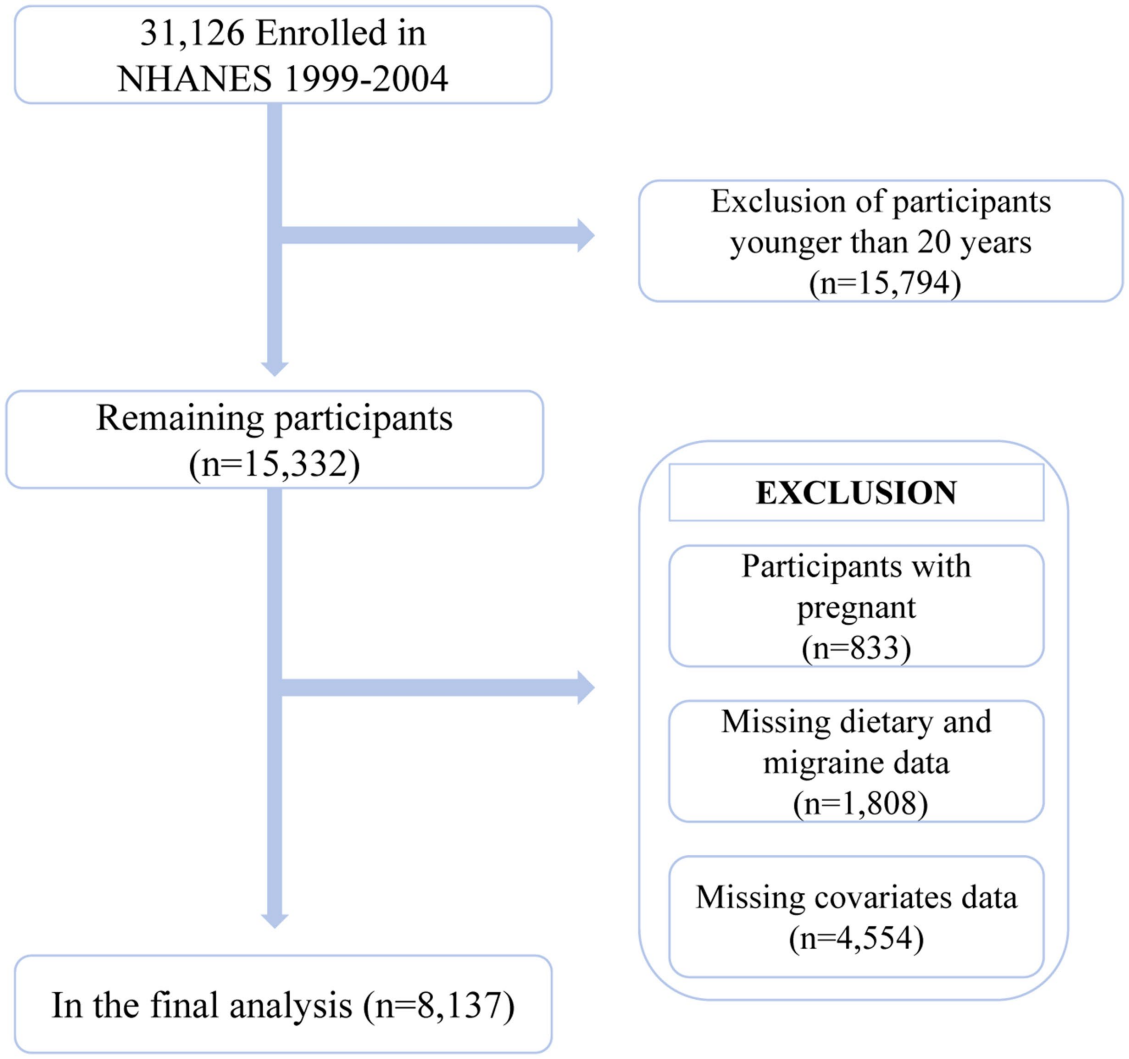
Flowchart of participant selection.

This is a cross-sectional study utilizing NHANES 1999–2004 data. NHANES is a large, nationally representative database compiled by the Centers for Disease Control and Prevention and the National Center for Health Statistics. It encompasses a wealth of information on demographics, social factors, and health-related data. All participants provided informed consent. The research design and methodological details of NHANES can be found on the official NHANES website.^1^ The 1999–2004 data collected from NHANES included information on migraine or severe headaches. A total of 31,126 participants across three cycles were initially included in the study. Subsequently, the following exclusions were made: (1) participants aged below 20 (*n* = 15,794); (2) pregnant participants (*n* = 833); (3) participants lacking data on the intake of six antioxidants in their diet (*n* = 1,788); (4) participants lacking data on migraine, severe headaches (*n* = 6), and sampling weights (*n* = 14); (5) participants with missing covariate data (*n* = 4,554). Overall, 8,137 participants were included in the data analysis (Figure 1).

### 2.2 Assessment of migraine

Our inclusion criteria for migraine patients were from the miscellaneous pain section of the NHANES self-assessment questionnaire. In Miscellaneous Pain Questionnaire (MPQ) 090, participants who answered “yes” to the question “During the past 3 months, did you experience severe headaches or migraine?” were defined as migraine patients (20–22).

### 2.3 CDAI

NHNAES dietary survey records were divided into two discontinuous recall information, collected at the mobile inspection center on the day of the interview and over the phone from days 3 to 10. These data included the types and quantities of all food and beverages consumed by participants 24 h before the interview. The method of calculating the intake of various nutrients by evaluating the average of two pieces of information has been proven to be more accurate than single data. Since there was only information from the first 24-h dietary review interview in NHANES 1999–2002, we also used the first 24-h recall data in NHANES 2003–2004 to maintain consistency and accuracy.

The intakes of six common dietary antioxidants were assessed, including vitamins A, C, and E, carotenoid, zinc, and selenium. After referring to the CDAI developed by Wright et al. (19) the daily intake of dietary antioxidants was normalized to estimate CDAI. To obtain a standardized CDAI, each antioxidant was standardized by subtracting the total mean, dividing the total standard deviation, and finally adding the resulting values.

### 2.4 Covariates

Based on the description of previous studies, the covariates were as follows. Demographic covariates were considered, including age, sex, and race. Race was categorized as Mexican-American, other Hispanic, non-Hispanic white, non-Hispanic black, and other races. Additionally, two socio-economic indicators were included, i.e., income level and educational attainment. The poverty-to-income ratio was divided into three groups (≤1, 1–3, and ≥3) for the assessment of income level. Educational level attainment was classified as less than high school, high school or equivalent, and higher than high school. Marital status was categorized as married/living with a partner, divorced/separated/widowed, and never married.

Health-related covariates included smoking status, drinking status, activity status, body mass index (BMI), and C-reactive protein (CRP). Smoking status was categorized as a non-smoker, former smoker, and current smoker. Drinking status was classified into three degrees according to the definition of the National Institute on Alcohol Abuse and Alcoholism: non-drinker, moderate drinker (1 drink per day for women or 1–2 drinks per day for men), and heavy drinker (≥2 drinks per day for women or ≥3 drinks per day for men). Activity status was divided into inactive, moderate exercise, and vigorous exercise. Body mass index (BMI) was grouped into four categories based on numerical values (underweight: <18.5, normal: 18.5–24.99, overweight: 25–29.99, obesity: ≥30). CRP was determined using the latex-enhanced turbidimetric method.

Additionally, several common diseases were included, namely hypertension, diabetes, coronary heart disease, and stroke. The diagnosis of these diseases was determined based on information provided by the patients to their physicians. Moreover, the diagnostic criteria for hypertension and medication usage. The diagnostic criteria for diabetes included a glycated hemoglobin level of ≥6.5% or fasting blood glucose of >126 mg/dL.

### 2.5 Statistical analysis

Specific weights, incorporating information on original sampling units, dietary weights, and stratum details were applied for the analysis. For dietary data from 1999–2002 and 2003–2004, dietary day one 4-year sample weight (WTDR4YR) and dietary day one sample weight (WTDRT1) were chosen, respectively. The calculation method was as follows: WTDR4YR × 4/6, WTDRT1 × 2/6. The selection of these weights was based on the analytical guidelines provided by NHANES and previous studies (16).

After data weighting, continuous variables were expressed as weighted means ± standard errors (SE), and categorical variables were presented as weighted percentages and frequencies. Weighted linear regression analysis was employed to compare between-group differences for continuous variables, while chi-square tests were used for categorical variables. Participants were categorized into four groups based on quartiles of the calculated CDAI, with the lowest quarter serving as the reference. The association between CDAI and migraine was assessed using logistic regression, with odds ratio (OR) and 95% confidence interval (95% CI) as measures of association. Subgroup analyses were conducted after stratification by age (20–50, >50 years) and sex (male, female). After grouped, logistic regression analysis was performed, incorporating all covariates to better understand the association between the two variables among different populations. To assess the robustness of the results, a sensitivity analysis was also conducted on participants without extreme energy intake, consuming <500 or >5,000 kcal per day. Finally, a restricted cubic spline (RCS) regression analysis was employed to investigate the nonlinear relationship between CDAI and migraine. CDAI was incorporated into the model as a continuous variable, adjusting for all potential covariates. A segmented logistic regression model was applied to determine the threshold of association and conduct threshold effect analysis.

The statistical analysis was conducted using STATA 17.0 (StataCorp LLC, College Station, Texas, United States), R software (version 4.3.1^2^; R Foundation for Statistical Computing, Austria), and EmpowerStats (X&Y Solutions, California, USA^3^). Bilateral *p*-values less than 0.05 were considered statistically significant.

## 3 Results

### 3.1 Baseline characteristics

The baseline characteristics of participants are depicted in Table 1. Overall, 8,137 participants from 1999 to 2004 NHANES were included in this analysis, of whom 1,610 individuals (19.98%) experienced migraine. Compared with non-migraine participants, individuals with migraine were more likely to be younger and female, had lower educational attainment, had lower to middle-income levels, had a higher BMI, were current smokers, heavy drinkers, and inactive, had no previous history of diabetes, and had a history of stroke, a lower protein intake, a higher carbohydrate intake, a higher CRP, a lower intake of most dietary antioxidants (except for vitamin E), and a lower CDAI.

**Table 1:** Characteristics of the study population from NHANES 1999–2004.

Characteristics	Overall (*n* = 8,137)	Non-migraine (*n* = 6,527)	Migraine (*n* = 1,610)	*p* value
Age (years, mean ± SE)	45.02 ± 0.32	46.11 ± 0.39	41.04 ± 0.39	<0.001
**Sex, *n* (%)**				<0.001
Male	4,252 (50.87%)	3,648 (54.67%)	604 (37.11%)	
Female	3,885 (49.13%)	2,879 (45.33%)	1,006 (62.89%)	
**Race, *n* (%)**				0.020
Mexican American	1,728 (6.73%)	1,358 (6.62%)	370 (7.16%)	
Other Hispanic	346 (4.98%)	265 (4.69%)	81 (6.05%)	
Non-Hispanic White	4,316 (74.59%)	3,535 (75.22%)	781 (72.29%)	
Non-Hispanic Black	1,485 (9.52%)	1,155 (9.19%)	330 (10.72%)	
Other race	262 (4.18%)	214 (4.29%)	48 (3.78%)	
**Education level, *n* (%)**				<0.001
Less than high school	4,224 (42.53%)	3,311 (40.55%)	913 (49.73%)	
High school or equivalent	2,227 (31.07%)	1,766 (31.13%)	461 (30.84%)	
More than high school	1,686 (26.40%)	1,450 (28.32%)	236 (19.43%)	
**Marital status, *n* (%)**				
Married/living with partner	5,097 (64.00%)	4,123 (64.30%)	974 (62.93%)	0.400
Divorced/separated/widowed	1,670 (17.48%)	1,336 (17.14%)	334 (18.70%)	
Never married	1,370 (18.52%)	1,068 (18.56%)	302 (18.37%)	
**Poverty to income ratio, *n* (%)**				<0.001
≤1	1,359 (12.76%)	991 (11.04%)	368 (19.01%)	
>1, <3	3,290 (34.14%)	2,604 (33.22%)	686 (37.47%)	
≥3	3,488 (53.10%)	2,932 (55.74%)	556 (43.52%)	
**Body mass index, *n* (%)**				<0.001
<18.5	118 (1.75%)	80 (1.41%)	38 (2.99%)	
18.5–24.99	2,544 (34.19%)	2,060 (34.52%)	484 (33.02%)	
25–29.99	2,975 (34.33%)	2,466 (35.43%)	529 (30.30%)	
≥30	2,500 (29.73%)	1,941 (28.64%)	559 (33.69%)	
**Smoking status, *n* (%)**				<0.001
Never	4,119 (50.11%)	3,292 (50.53%)	827 (48.57%)	
Former	2,064 (24.00%)	1,766 (25.33%)	298 (19.17%)	
Current	1,954 (25.89%)	1,469 (24.14%)	485 (32.26%)	
**Drinking status, *n* (%)**				<0.001
Non-drinkers	1,358 (14.35%)	1,057 (13.95%)	301 (15.82%)	
Moderate drinkers	2,862 (35.84%)	2,372 (37.07%)	490 (31.35%)	
Heavy drinkers	3,917 (49.81%)	3,098 (48.98%)	819 (52.83%)	
**Activities status, *n* (%)**				<0.001
Inactive	3,203 (32.14%)	2,508 (31.15%)	695 (35.75%)	
Moderate	2,335 (30.39%)	1,899 (30.28%)	436 (30.75%)	
Vigorous	947 (11.95%)	754 (11.68%)	193 (12.94%)	
Both	1,652 (25.52%)	1,366 (26.88%)	286 (20.56%)	
**Hypertension, *n* (%)**				0.227
No	5,659 (73.66%)	4,528 (73.97%)	1,131 (72.53%)	
Yes	2,478 (26.34%)	1,999 (26.03%)	479 (27.47%)	
**Diabetes, *n* (%)**				<0.001
No	7,172 (91.8%)	5,725 (91.26%)	1,447 (93.77%)	
Yes	965 (8.2%)	802 (8.74%)	163 (6.23%)	
**Stroke, *n* (%)**				0.001
No	7,916 (97.87%)	6,359 (98.14%)	1,557 (96.91%)	
Yes	221 (2.13%)	168 (1.86%)	53 (3.09%)	
**Coronary heart disease, *n* (%)**				0.063
No	7,809 (96.64%)	6,245 (96.45%)	1,564 (97.35%)	
Yes	328 (3.36%)	282 (3.55%)	46 (2.65%)	
C-reactive protein (mg/dl, mean ± SE)	0.39 ± 0.01	0.38 ± 0.01	0.43 ± 0.02	0.008
Energy (kcal/day, mean ± SE)	2249.09 ± 16.88	2252.42 ± 20.57	2236.97 ± 36.42	0.584
Protein intake (g/day, mean ± SE)	83.49 ± 0.72	84.40 ± 0.76	80.17 ± 1.43	<0.001
Carbohydrate intake (g/day, mean ± SE)	275.87 ± 2.50	274.21 ± 2.89	281.90 ± 5.46	0.040
Vitamin A intake (mcg/day, mean ± SE)	722.38 ± 18.44	749.01 ± 20.67	625.69 ± 24.16	<0.001
Carotenoid intake (mcg/day, mean ± SE)	1916.43 ± 78.75	1989.64 ± 91.23	1650.6 ± 155.75	0.003
Vitamin C intake (mcg/day, mean ± SE)	93.81 ± 2.86	96.04 ± 2.79	85.70 ± 4.49	<0.001
Vitamin E intake (mcg/day, mean ± SE)	8.00 ± 0.11	8.04 ± 0.13	7.85 ± 0.20	0.309
Zinc intake (mg/day, mean ± SE)	12.12 ± 0.15	12.25 ± 0.15	11.63 ± 0.27	0.004
Selenium intake (mcg/day, mean ± SE)	0.39 ± 0.01	112.63 ± 1.27	104.9 ± 1.98	<0.001
CDAI (mean ± SE)	0.26 ± 0.08	0.37 ± 0.09	−0.13 ± 0.12	<0.001

Mean ± SE for continuous variables: *p*-value was calculated by weighted linear regression.

Unweighted number (weighted percentage) for categorical variables: *p*-value was calculated by weighted chi-square test. *p* < 0.05 was considered statistically significant.

SE, Standard error; CDAI, Composite dietary antioxidant index.

### 3.2 Association between CDAI and migraine

Logistic regression revealed a significant negative association between CDAI and migraine (Table 2). This result remained significant in Model 3 after adjusting for all covariates. Compared with participants in the lowest CDAI quartile (Q1: −5.83 to −2.14), adjusted OR for participants in Q3 (−0.59 to 1.53) and Q4 (1.53–44.63) quartiles were 0.71 (95% CI: 0.54–0.92, *p* = 0.011) and 0.64 (95% CI: 0.47–0.87, *p* = 0.005), respectively. After excluding participants with extreme energy intake, 7,929 participants remained, and the relationship between CDAI and migraine remained stable. Compared with individuals with the lowest CDAI quartile (Q1: −5.77 to −2.13), adjusted OR for CDAI and migraine in Q3 (−0.62 to 1.43) and Q4 (1.43–44.63) quartiles were 0.70 (95% CI: 0.54–0.90, *p* = 0.008) and 0.62 (95% CI, 0.46–0.85, *p* = 0.004), respectively (Table 3).

**Table 2:** Association between CDAI and migraine.

CDAI (quintiles)	No.	Model 1 OR (95% CI)	*p* value	Model 2 OR (95% CI)	*p* value	Model 3 OR (95% CI)	*p* value
Q1 (−5.83 to −2.14)	2,034	1.00 (Reference)		1.00 (Reference)		1.00 (Reference)	
Q2 (−2.14 to −0.59)	2,034	0.91 (0.73, 1.14)	0.413	0.97 (0.78, 1.21)	0.761	0.96 (0.76, 1.21)	0.715
Q3 (−0.59 to 1.53)	2,034	0.65 (0.52, 0.81)	<0.001	0.74 (0.59, 0.94)	0.013	0.71 (0.54, 0.92)	0.011
Q4 (1.53–44.63)	2,035	0.61 (0.50, 0.75)	<0.001	0.74 (0.60, 0.90)	0.004	0.64 (0.47, 0.87)	0.005
P for trend			0.001		0.066		0.257

Model 1: unadjusted.

Model 2: adjusted for age, sex and race.

Model 3: adjusted all covariates, including that age, sex, race, education level, marital status, poverty to income ratio, body mass index, smoking status, drinking status, activities status, hypertension, diabetes, stroke, coronary heart disease, C-reactive protein, energy, protein intake and carbohydrate intake.

CDAI, composite dietary antioxidant index; OR, odds ratio; CI, confidence interval.

**Table 3:** Association between CDAI and migraine without extreme energy intake individuals.

CDAI (quintiles)	No.	Model 1 OR (95% CI)	*p* value	Model 2 OR (95% CI)	*p* value	Model 3 OR (95% CI)	*p* value
Q1 (−5.77 to −2.13)	1,982	1.00 (Reference)		1.00 (Reference)		1.00 (Reference)	
Q2 (−2.13 to −0.62)	1,982	0.93 (0.74, 1.16)	0.510	0.98 (0.79, 1.23)	0.890	0.96 (0.77, 1.20)	0.709
Q3 (−0.62 to 1.43)	1,982	0.68 (0.53, 0.82)	<0.001	0.76 (0.60, 0.95)	0.020	0.70 (0.54, 0.90)	0.008
Q4 (1.43–44.63)	1,983	0.60 (0.49, 0.75)	<0.001	0.73 (0.59, 0.90)	0.004	0.62 (0.46, 0.85)	0.004
P for trend			0.001		0.029		0.102

Model 1: unadjusted.

Model 2: adjusted for age, sex and race.

Model 3: adjusted all covariates, including that age, sex, race, education level, marital status, poverty to income ratio, body mass index, smoking status, drinking status, activities status, hypertension, diabetes, stroke, coronary heart disease, C-reactive protein, energy, protein intake and carbohydrate intake.

CDAI, composite dietary antioxidant index; OR, odds ratio; CI, confidence interval.

### 3.3 Subgroup analysis

**Figure 2:**
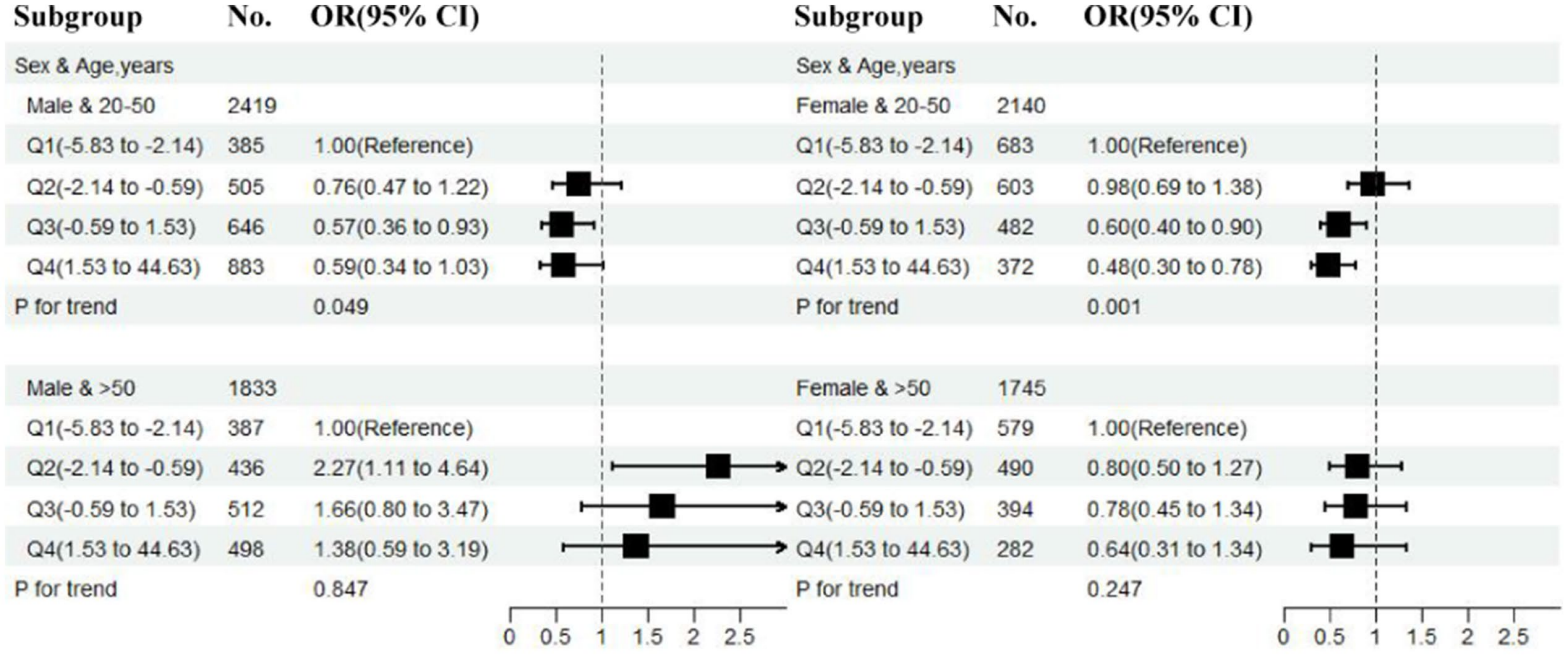
Relationship of CDAI on migraine in different subgroup (stratified by sex and age). Except the stratification variables, all other variables will be included (race, education level, marital status, poverty to income ratio, body mass index, smoking status, drinking status, activities status, hypertension, diabetes, stroke, coronary heart disease, C-reactive protein, energy, protein intake, and carbohydrate intake). CDAI, composite dietary antioxidant index; OR, odds ratio; CI, confidence interval.

To further explore the potential association between CDAI and migraine, participants were stratified based on age (20–50, >50 years) and sex (male, female). The results are presented in Figure 2. Compared with the other three subgroups (males aged 20–50, males and females aged >50), a highly significant negative association was observed between CDAI and migraine in females aged 20–50 group. In the logistic regression model, compared with the reference group Q1 (−5.83 to −2.14), the adjusted OR gradually decreased with an increase in CDAI. Specifically, the OR was 0.60 (95% CI: 0.40–0.90, *p* = 0.013) for Q3 (−0.59 to 1.53), whereas the risk was even reduced by more than half for Q4 (1.53–44.63), with the OR of 0.48 (95% CI: 0.30–0.78 *p* = 0.003). However, such significant negative associations were hardly observed in other subgroups, except for the Q3 group (−0.59 to 1.53) in males aged 20–50, in which the adjusted OR was 0.57 (95% CI: 0.36–0.93, *p* = 0.023).

**Figure 3:**
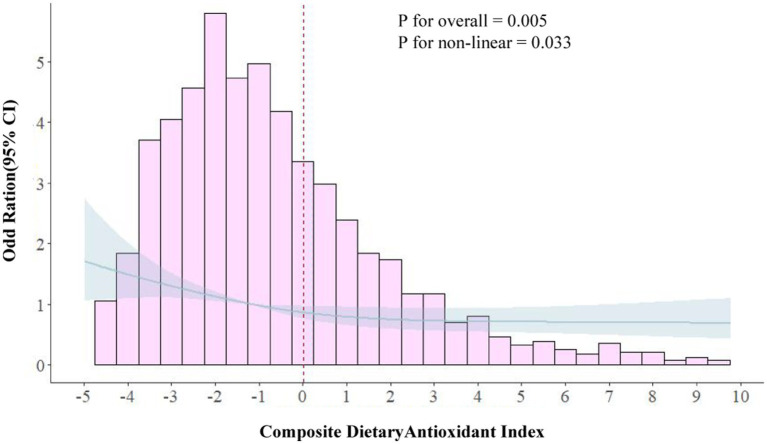
Association between composite dietary antioxidant index and migraine in 20–50 females in RCS. The model was adjusted for age, race, education level, marital status, poverty to income ratio, body mass index, smoking status, drinking status, Activities status, hypertension, diabetes, stroke, coronary heart disease, C-reactive protein, energy, protein intake and carbohydrate intake. CDAI, composite dietary antioxidant index; OR, odds ratio.

The RCS curve results demonstrated a nonlinear relationship between CDAI and migraine in the subgroup of females aged 20–50 (Figure 3). The *p*-value for nonlinear was <0.001, indicating an L-shaped curve. In the threshold analysis, the adjusted OR for migraine was 0.86 (95% CI: 0.78–0.93, *p* < 0.001) in participants with a CDAI of <0.006. This implied that the risk of migraine was decreased by 14% with every 1 unit increase in CDAI. Meanwhile, CDAI was negatively associated with migraine when the CDAI was >0.006 but the association was not statistically significant (Table 4). This suggested that the risk of migraine did not increase with a CDAI of >0.006 in this subgroup. Collectively, these data indicate that the protective effect of CDAI on migraine attacks may not be as good as before after the threshold point.

**Table 4:** Threshold effect analysis of the association of CDAI with migraine in female with 20–50 years.

CDAI	Adjust OR (95% CI)	*p* value
<0.006	0.86 (0.78–0.93)	<0.001
>0.006	0.98 (0.93–1.03)	0.469
P for likelihood ratio test		0.008

Fully adjusted for age, sex race, education level, marital status, poverty to income ratio, body mass index, smoking status, drinking status, activities status, hypertension, diabetes, stroke, coronary heart disease, C-reactive protein, energy, protein intake and carbohydrate intake.

CDAI, composite dietary antioxidant index; OR, odds ratio; CI, confidence interval.

## 4 Discussion

The current cross-sectional study based on NHANES data provides nationally representative conclusions, demonstrating a negative correlation between CDAI and migraine in the adult population of the United States. This association is independent of other confounding factors. Moreover, simultaneous stratification of age and sex revealed a strengthened protective effect of CDAI against migraine in adult females aged below 50 years. Additionally, a clear nonlinear relationship was observed between CDAI and migraine, indicating the presence of a threshold effect.

Over the past decade, vitamins C and E have been widely recognized as effective antioxidants with positive implications for the prevention and treatment of migraine (9, 23, 24). However, a previous study reported that the intake of complex vitamins containing vitamins C and B and algae may increase the risk of migraine in women (25). Another study indicated that supplementing with vitamin C could reduce OS but might only be effective in individuals with initially low vitamin C concentrations (26). This may be one of the reasons why the effectiveness of vitamin C supplements varies among different populations. These contradictory results warrant further validation using randomized controlled trials. Vitamin E is frequently mentioned in the context of menstrual migraine. A study instructed female migraine patients to take 400 IU of vitamin E daily for 5 days before and after menstruation, which resulted in a significant improvement in migraine symptoms after three cycles (23). This effect may be associated with the inhibitory action of vitamin E on prostaglandin (PG). It has been reported that PG can be released in large quantities before menstruation, and vitamin E can inhibit the release of arachidonic acid by acting on phospholipase A2 and cyclooxygenase, preventing its conversion to PG (27). In the dietary antioxidant intake data included in the present study, only vitamin E showed no significant difference between the two comparison groups. However, when standardized to the CDAI, it exhibited a significant protective effect against migraine. It is believed that this may be related to the criteria for variable selection and elimination, indicating that antioxidants are likely to act collaboratively rather than relying on a single component. Chayasirisobhon reported a study involving 12 patients with a long history of migraine and ineffective treatment with various medications (such as beta-blockers and serotonin receptor agonists). These patients were instructed to take a combination capsule containing pine bark extract and a combination of vitamins C and E for three consecutive months. The results showed a significant improvement in headache duration and severity, along with a notable reduction in migraine disability scores (28). These findings align closely with our conclusions.

Increased intake of trace elements also exhibits a protective effect against migraine; however, there is limited literature on the direct mechanistic actions of zinc and selenium in migraine. Although zinc is not a direct antioxidant, at physiological concentrations it can indirectly exert antioxidant effects through certain pathways. For instance, zinc can bind to the sulfate donor of cysteine, and the interaction between oxidants and sulfide donors releases free zinc. The oxidation-induced release of zinc from the cysteine residue sulfate donor generates a zinc signal, triggering antioxidant responses against ROS and OS (29–31). Zinc possesses anti-inflammatory properties and may alleviate headaches by reducing proinflammatory cytokines (32). Zinc is also a component of superoxide dismutase (SOD), and its deficiency can hinder SOD synthesis, leading to OS (29). Two cross-sectional studies from NHANES reported a negative correlation between dietary zinc intake in American adults and migraine, consistent with our findings (16, 22). The contribution of selenium to antioxidant defense is diverse. As the most abundant selenium protein, glutathione peroxidase 1 is the primary metabolic form of selenium in the body combating severe OS, primarily by counteracting ROS to maintain redox balance. A meta-analysis reported that selenium supplementation may reduce OS by increasing total antioxidant capacity, glutathione peroxidase, and decreasing plasma malondialdehyde (33). However, there is a dearth of reports on the potential mechanisms of protective effects of selenium in patients with migraine, despite some studies confirming differences in selenium levels between migraine and non-migraine patients (17, 34). Nazıroğlu and colleagues discovered in an animal model that selenium supports the antioxidant system by inhibiting free radicals and modulating the activity of mitochondrial membrane Ca(2+)-ATPase to protect headache-induced oxidative toxicity in rats exposed to nitroglycerin (35). This may represent one of the potential mechanisms.

Carotenoids are important precursors of vitamin A. Although there are currently few direct reports on their association with migraine, the potential mechanisms of their improvement in migraine can be explored through existing theories. β-carotene is the most important carotenoid in the human body and an effective fat-soluble antioxidant that maintains a reducing microenvironment in biological systems. It possesses direct antioxidant activities, including the ability to scavenge singlet oxygen and peroxyl radicals, and enhances antioxidant levels (such as glutathione and SOD) (36, 37). In contrast to β-carotene, the antioxidant action of vitamin A is indirect. All-trans-retinoic acid, a metabolite of vitamin A, acts as a ligand-dependent transcription factor regulating the expression levels of a series of target genes necessary for the body to generate effective antioxidant responses (36). The involved pathways, such as the Toll-like receptor 4 and 5′AMP-activated protein kinase signaling pathway, are crucial antioxidant regulatory systems in the body (38). In summary, our study supplements the role of these nutrients in migraine. However, the independent impact of a single nutrient on migraine prevention does not imply a direct physiological correlation with migraine attacks for all included nutritional factors. Generally, it is impossible to intake only a specific nutrient in real-world dietary patterns. Therefore, several studies have also recognized the value of CDAI, such as its protective effects against depression, cardiovascular diseases, and muscle preservation (10, 18, 39). Considering the complex pathophysiological mechanisms of migraine, more research is still needed to explore the interaction mechanisms of various antioxidants.

Furthermore, we have derived practical conclusions from our subgroup analysis. Currently, researchers widely believe that hormonal fluctuations are among the most significant factors contributing to the differences in migraine attacks between men and women. As women age, the onset of migraine gradually increases from adolescence. Most women experience an improvement in the frequency and/or severity of migraine after pregnancy and menopause, closely aligning with the trends in estrogen changes (40). Notably, estrogen can exert a profound impact on the mitochondrial respiratory chain by regulating mitochondrial gene transcription. Simultaneously, substantial accumulation of estrogen can also trigger the excessive generation of ROS, further exacerbating mitochondrial DNA (mtDNA) mutations and damage to mitochondrial proteins. Compared with histone-protected nuclear DNA, mtDNA is more susceptible to the impact of OS (41). These findings support our conclusions regarding age and gender grouping and underscore the importance of increased dietary antioxidant intake, especially in adult women aged below 50 years. Menstrual migraine is a special condition associated with a decrease in estrogen, but taking low-dose estrogen to treat migraine may also increase the risk of other diseases. This also reflects the potential of increasing antioxidant capacity through diet (12).

The present study explored the potential association between CDAI and migraine based on NHANES data, identifying high-risk populations with research value. Previous research has suggested a significant negative association between overall dietary antioxidant capacity and migraine attack frequency; however, CDAI showed no significant association with migraine (42). Building on these findings, our study leveraged the comprehensive survey approach of the NHANES system and included a much larger sample size than previous studies. Additionally, we incorporated more variables that could potentially impact the results. The conclusions drawn from this study provide valuable insights into the prevention and treatment of migraine.

Nonetheless, this study has several limitations. Firstly, this is a cross-sectional study with a relatively long period. While the use of an average of two 24-h dietary recalls has distinct advantages, it is not completely scientifically accurate and is susceptible to bias. Secondly, the pathophysiological mechanisms of migraine are highly complex, and the body’s antioxidant system cannot solely rely on one or a few substances for maintenance. There may also be confounding factors not included in the study, such as certain chronic diseases, which may have important potential impacts on the conclusions. Third, this study specifically analyzed American adults, and the findings may not apply to other ethnicities, populations, or minors. Finally, differences in consumption and dietary patterns between the time of data collection (1999–2004) and the present should be considered. Therefore, targeted prospective research is highly necessary. Future studies may build on these nutritional substances as a foundation and gradually expand the variety of dietary antioxidants until an optimal combination is identified.

## 5 Conclusion

In summary, the current study demonstrates that high levels of CDAI are significantly negatively correlated with migraine attacks. This protective effect is particularly pronounced in females aged 20–50. Therefore, increasing the intake of dietary antioxidants may have some value in reducing the risk of migraine attacks and occurrence.

## Data Availability

The original contributions presented in the study are included in the article/Supplementary material, further inquiries can be directed to the corresponding author.

## Footnotes

^1^https://www.cdc.gov/nchs/nha-nes/index.htm

^2^http://www.R-project.org

^3^http://www.empowerstats.com
